# P-497. Performance of the Brief Patient Health Questionnaire-2 and Generalized Anxiety Disorder-2 Screeners for Identifying Depression and Anxiety in People with HIV

**DOI:** 10.1093/ofid/ofae631.696

**Published:** 2025-01-29

**Authors:** Ye Eun Lee, Maria C Latimer, Sharon Kelly, Tarfa I Verinumbe, Tracy Agee, Heidi Hutton, Joyce L Jones, Sheree R Schwartz, Jeffrey H Hsu, Oluwaseun Falade-Nwulia

**Affiliations:** Johns Hopkins University School of Medicine, Baltimore, Maryland; Johns Hopkins University School of Medicine, Baltimore, Maryland; Johns Hopkins University School of Medicine, Baltimore, Maryland; Johns Hopkins University, Baltimore, Maryland; Johns Hopkins University, Baltimore, Maryland; Johns Hopkins Univ School of Medicine, Baltimore, Maryland; Johns Hopkins University School of Medicine, Baltimore, Maryland; Johns Hopkins School of Public Health, Baltimore, Maryland; Johns Hopkins University School of Medicine, Baltimore, Maryland; Johns Hopkins University, Baltimore, Maryland

## Abstract

**Background:**

Mental health disorder (MHD) prevalence is high among people with HIV (PWH) and linked to poor health outcomes, including HIV non-suppression. Despite recommendations for routine mental health screening in HIV care settings, screening uptake remains low. The brief Patient Health Questionnaire-2 (PHQ-2) and Generalized Anxiety Disorder-2 (GAD-2) are validated screeners for depression and anxiety symptoms with potential for integration into HIV care. This study evaluates the accuracy of the PHQ-2 and GAD-2 in detecting depression and anxiety among PWH and co-occurring MHD or substance use disorders (SUD).

**Figure:**
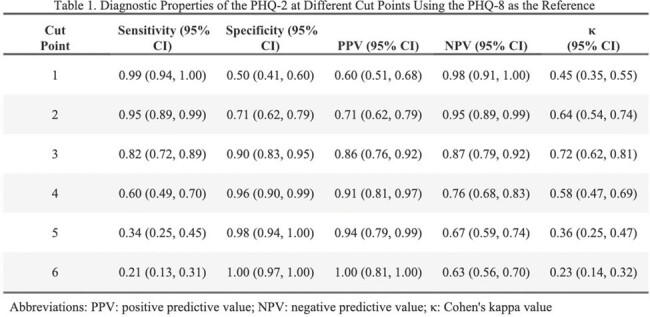

**Methods:**

Routine screening using the PHQ-2, GAD-2, and NIDA Quick Screen for SUD was implemented in an HIV care setting in Baltimore in April 2022. Patients who screened positive on any screener and enrolled in a study evaluating an intervention for SUD and MHD care were included in the analysis. Participants completed the longer PHQ-8 and GAD-7 screeners at enrollment, which served as reference standards using recommended cut points of ≥10 to indicate moderate or severe depression or anxiety, respectively. PHQ-2 and GAD-2 scores are derived from the first two questions of their corresponding longer measure. Area under the curve (AUC), sensitivity, and specificity were obtained for the PHQ-2 and GAD-2 at various cut points against their corresponding longer measure.

**Figure:**
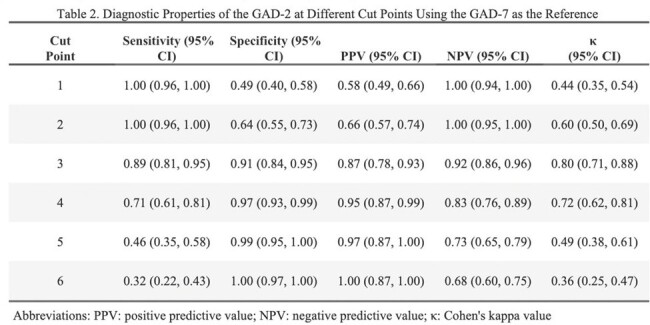

**Results:**

Of the 204 patients included in this study, 68% were male, 80% were Black, and mean age was 51 years. Compared to the PHQ-8 using a cut point of ≥10, the PHQ-2 at the cut point of ≥3 demonstrated high accuracy in detecting depression at a cut point of ≥3 (AUC = 0.86; 95% CI: 0.81, 0.91) with a sensitivity of 0.82 (95% CI: 0.72, 0.89) and specificity of 0.90 (95% CI: 0.83, 0.95). Compared to the GAD-7 using a cut point of ≥10, the GAD-2 was highly accurate in identifying anxiety at a cut point of ≥3 (AUC = 0.90; 95% CI: 0.86, 0.94), achieving a high sensitivity (0.89; 95% CI: 0.81, 0.95) and specificity (0.91; 95% CI: 0.84, 0.95).

**Conclusion:**

In the context of HIV care in busy clinical settings, our findings indicate that implementation of the PHQ-2 and GAD-2 screeners accurately detect depression and anxiety symptoms at the optimal cut point of ≥3. Simplified identification and treatment of comorbid MHD may improve HIV outcomes.

**Disclosures:**

**Oluwaseun Falade-Nwulia, MBBS ,MPH**, Abbvie Inc: Grant/Research Support|Gilead Sciences: Advisor/Consultant

